# The Effect of Terbinafine and Its Ionic Salts on Certain Fungal Plant Pathogens

**DOI:** 10.3390/molecules28124722

**Published:** 2023-06-12

**Authors:** Tao Wang, Qiuxiao Wang, Yifei Zhou, Yaolin Shi, Haixiang Gao

**Affiliations:** 1 Department of Applied Chemistry, College of Science, China Agricultural University, Beijing 100193, China; wangtao978@cau.edu.cn (T.W.); bs20203100716@cau.edu.cn (Q.W.); zhouyifei@cau.edu.cn (Y.Z.); 2 College of Materials Science and Engineering, Beijing University of Chemical Technology, Beijing 100029, China; 2020020050@buct.edu.cn; 3 Innovation Center of Pesticide Research, Department of Applied Chemistry, China Agricultural University, Beijing 100193, China

**Keywords:** terbinafine, ionic salts, solubility, surface tension, agricultural antifungal activity

## Abstract

Terbinafine, an inhibitor of squalene epoxidase in ergosterol biosynthesis, is chiefly utilized as an antifungal medication with potential uses in pesticide applications. This study explores the fungicidal efficacy of terbinafine against prevalent plant pathogens and confirms its effectiveness. To augment its water solubility, five ionic salts of terbinafine were synthesized by pairing them with organic acids. Among these salts, TIS 5 delivered the most impressive results, amplifying the water solubility of terbinafine by three orders of magnitude and lessening its surface tension to facilitate better dispersion during spraying. The in vivo experiments on cherry tomatoes showed that TIS 5 had a superior therapeutic activity compared to its parent compound and two commonly used broad-spectrum fungicides, pyraclostrobin and carbendazim. The results highlight the potential of terbinafine and its ionic salts, particularly TIS 5, for use as fungicides in agriculture due to their synergistic effects with furan-2-carboxylate.

## 1. Introduction

In the field of contemporary agriculture, the utilization of pesticides significantly contributes to the enhancement of crop yield and quality [1]. However, incessant pesticide application has resulted in an escalating resistance in pests and weeds. To address this decline in pesticide effectiveness, the development of new pesticides is essential. The connection between protein-targeting drugs, pesticides, and pharmaceuticals is intimate due to the commonality in their active functional groups. The structural similarity of the key components responsible for their effects varies depending on the specific target of action. For example, the antifungal agents such as fluconazole, itraconazole, and voriconazole, used in clinical settings, were developed based on lanosterol 14 α-demethylase (CYP51) during ergosterol biosynthesis [2]. Likewise, fungicides such as miconazole, triadimefon, and tebuconazole, which employ the same mechanism of action [3], have been developed. These compounds, known as azole antifungals, exert their effects through the competitive inhibition of CYP51. The presence of crucial active functional groups in both pharmaceuticals and pesticides targeting similar proteins have broad implications for the development of both pharmaceuticals and pesticides.

Terbinafine, an allylamine antifungal medication, is predominantly employed as a fungicide for treating skin fungi by inhibiting squalene epoxidase, thereby preventing the generation of ergosterol in fungal cell membranes [4]. Its safety profile in humans is enhanced due to its high selectivity for fungal enzymes, which minimally impacts cholesterol synthesis in mammals. Despite its potential as an agrofungicide and its non-toxicity to humans, terbinafine has not yet been developed for pesticide applications. Theoretically, terbinafine shows promise as an agrofungicide due to its non-toxicity to humans, marking a distinct advantage over existing alternatives. Some studies have shown that terbinafine effectively inhibits the growth of important plant pathogenic fungi at low concentrations [5,6]. Moreover, terbinafine has a low risk of resistance development in fungi due to its targeting of squalene epoxidase, which is less prone to mutations than other fungal enzymes [7]. Nonetheless, the research on the antifungal activity of terbinafine against plant pathogens is limited and dispersed. Only a few studies have reported on the inhibitory effects of terbinafine on certain plant pathogenic fungi, such as *Fusarium* spp., *Rhizoctonia solani,* and *Sclerotinia sclerotiorum* [5,6]. Moreover, most of these studies were conducted in vitro, and the in vivo efficacy and safety of terbinafine on crops and plants have not been well investigated. Therefore, there is a need for more comprehensive and systematic studies on the agricultural antifungal activity of terbinafine and its potential applications as a novel agrofungicide.

The conversion of a drug into its salt form can augment its activity and refine its physical and chemical properties, which is applicable to both pesticides and medications [8,9]. For instance, ketoconazole citrate and tartrate display an enhanced solubility in buffer solutions compared to their parent, ketoconazole [10]. In the realm of pesticides, the combination of myclobutanil and thiabendazole with a docusate anion results in a salt with an improved rain persistence and a superior protective activity against potato tuber diseases [11]. Additionally, pyrimethanil 2-hydroxybenzoate demonstrates reduced volatilization and photodegradation compared to its parent pyrimethanil, thus mitigating its environmental impact [12]. The storage stability of cyprodinil is also enhanced by eliminating cyprodinil polymorphs in its salt form [13]. Terbinafine, containing a tertiary amine in its structure and a pKa of 7.12 [14], is highly esterophilic and may exhibit poor water solubility. However, its pKa value enables it to form salts with certain organic acids.

The goal of this research was to examine the feasibility of employing terbinafine as a pesticide. In pursuit of this, its fungicidal capacity against agropathogenic fungi was assessed and then five salts of terbinafine were prepared via acid–base neutralization reactions to optimize its physical and chemical properties. The physical and chemical characteristics (including the melting point, water solubility, surface activity, etc.) and the fungicidal activity of terbinafine and its ionic salts (TISs) were subsequently analyzed.

## 2. Results and Discussion

### 2.1. Salt Preparation

This research aims to synthesize five terbinafine ionic salts (TISs) using different acids. Before presenting the synthetic pathway, the selection criteria for the acids and their implications for the properties of the TISs were discussed. Five acids were chosen for this study: salicylic acid, chlorendic acid, 2-furoic acid, maleic acid, and fumaric acid [15,16,17,18,19,20,21].

The main criterion for selecting these acids was their compatibility with terbinafine to form ionic salts. Ionic salt formation, in this context, bears two fundamental implications: an enhancement in water solubility and a reduction in the surface tension of terbinafine. A higher water solubility leads to a better bioavailability of the drug, which improves its effectiveness. Concurrently, the reduction in the surface tension facilitates a superior dispersion during application via aerosolized routes [22,23,24]. 

These acids were selected based on both their chemical and biological properties. Notably, salicylic acid and chlorendic acid demonstrated significant antifungal attributes in prior investigations [16,19]. Furthermore, these acids have been used, both individually and in combination, against an array of phytopathogens, thus indicating their potential efficacy in such applications [17]. 

In the current study, the aim was to contribute further to the existing scientific discourse on this subject. The information extracted from our research resonated with the findings of previous studies regarding the antifungal effectiveness of these acids. Therefore, enhancing the properties of terbinafine through ionic salt formation might imply a synergistic or contributory effect. However, such an assertion necessitates additional rigorous scientific scrutiny to substantiate this hypothesis.

**Scheme 1:**
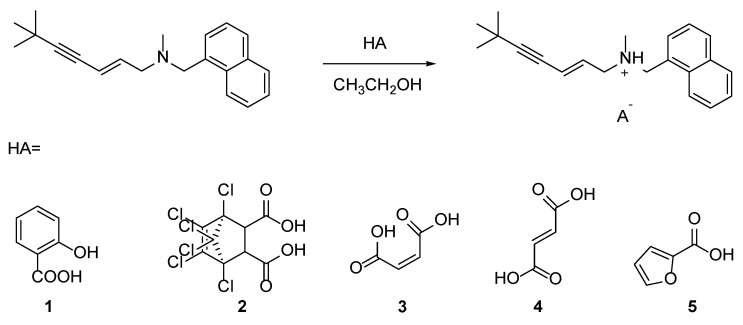
Synthesis of TISs and the structures of anions.

Five terbinafine ionic salts (TISs) were prepared by reacting terbinafine with different organic acids in a 1:1 molar ratio in ethanol, as shown in Scheme 1. The solvent was then evaporated, followed by the addition of petroleum ether dropwise to the oily residue. The polar ionic salt precipitated in the non-polar solvent, and it was filtered and washed with the petroleum ether, resulting in TIS 1, TIS 3, and TIS 5 respectively. TIS 2 precipitated as a solid during the reaction and then it was washed with ethanol and dried. The evaporation of a mixture of terbinafine and fumaric acid resulted in a white crude product, which was then washed with petroleum ether and filtered to obtain TIS 4. TIS 1, TIS 2, TIS 3, and TIS 4 had melting points above 100 °C, while TIS 5 had a melting point below 100 °C and could be considered an ionic liquid. The products were characterized using NMR spectroscopy and IR spectroscopy. Table 1 shows the chemical shift of three types of hydrogen atoms attached to the carbon of the tertiary amine. Compared to terbinafine, these hydrogen atoms in the terbinafine salts shifted from high field to low field regions in the NMR spectra, indicating the successful synthesis of the TISs.

### 2.2. Solubilities

Table 2 shows the solubility of terbinafine and its ionic salts (TISs) in different solvents. Terbinafine had a low solubility in water but a high solubility in various polar solvents, reaching 0.1 g/mL. TIS 1 and TIS 5 had a similar solubility to terbinafine, with a low water solubility and a high solubility in other polar solvents. However, TIS 2, TIS 3, and TIS 4 had a low solubility in most solvents, which could be due to their dicarboxylic acid anions. Among these three salts, TIS 2 had the lowest solubility, possibly due to its larger anion structure. This also explained why TIS 2 precipitated quickly in ethanol during the reaction. All the salts had a low solubility in hexane, a non-polar liquid. This confirmed that most salts were incompatible with low-polarity liquids.

**Figure 1:**
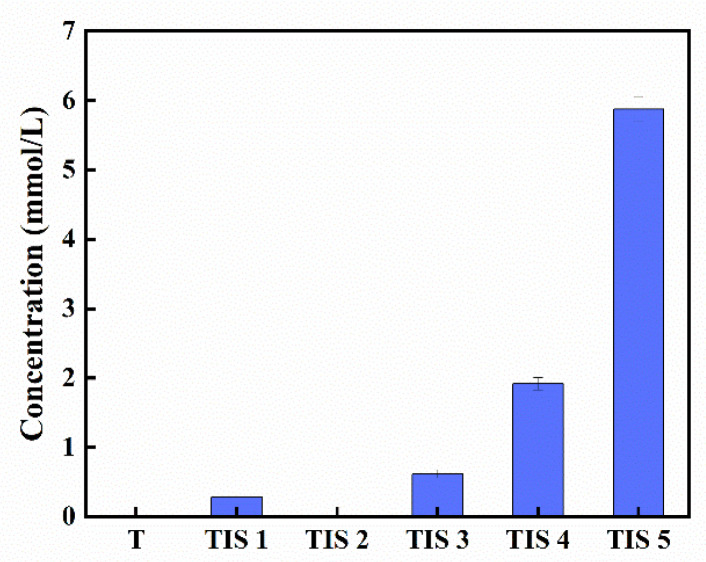
The solubilities of terbinafine and TISs in water at 25 °C.

The aqueous solubility of terbinafine and its ionic salts (TISs) were examined and the results are presented in Table S1 and Figure 1. Terbinafine showed a notably low water solubility (5.22 μmol/L), presumably due to the hydrophobic nature of the cations in its salts. The poor solubility of all the prepared TISs in water was attributed to this same factor. Nonetheless, the water solubility of the TISs improved significantly, ranging from 5.62 μmol/L to 5.88 mmol/L. Specifically, TIS 4 (1.92 mmol/L) and TIS 5 demonstrated a three orders of magnitude greater solubility than terbinafine alone. Importantly, TIS 5 achieved the highest solubility of all the TISs, reaching 5.88 mmol/L. This outcome aligned with the expectation that forming terbinafine salts would enhance water solubility, suggesting a potential usefulness of these compounds as fungicides in agricultural applications.

### 2.3. Surface Activity

**Figure 2:**
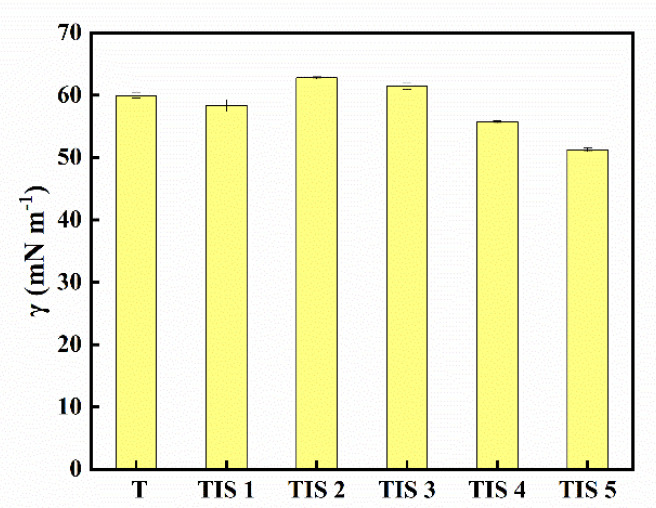
The surface tensions of the saturated aqueous solutions of terbinafine and TISs at 25 °C.

The surface tensions of the saturated aqueous solutions of terbinafine and its ionic salts (TISs) at 25 °C are presented in Figure 2. The surface tension of TIS 2 was found to be the highest, with a value of 62.78 mN·m^−1^, followed by TIS 3 (61.48 mN·m^−1^), terbinafine (T: 59.92 mN·m^−1^), TIS 1 (58.33 mN·m^−1^), TIS 4 (55.68 mN·m^−1^), and TIS 5 (51.26 mN·m^−1^). The results indicated that the anions in TIS 2 and TIS 3 did not effectively reduce the surface tension. In general, the solutions with a lower surface tension exhibited longer retention times and an increased coverage of the pesticide on the plant, thereby enhancing its bioactivity [12,25]. The surface tension of the TIS 5 solution was the smallest among the TISs and was lower than that of the parent terbinafine solution. Therefore, TIS 5 was expected to exhibit a superior biological activity among the TISs. 

The critical micelle concentrations (CMCs) of the TISs were measured using electrical conductivity at 25 °C, as shown in Figure S16. TIS 4 and TIS 5 had measurable CMCs of 0.31 mmol/L and 0.66 mmol/L, respectively, indicating that they could form micelles at these concentrations. However, the CMCs of TIS 1, TIS 2, and TIS 3 could not be measured because they were probably insoluble in water under ambient conditions, which prevented them from forming micelles. Therefore, reliable data on the CMCs of these compounds were not available.

### 2.4. In Vitro Assays of Fungicidal Activities

The antifungal activities of terbinafine and its ionic salts (TISs) in vitro was evaluated using the mycelial growth inhibition method with 50 mg/L solutions. Pyraclostrobin, a commercial fungicide, was used as a positive control. Table 3 shows that terbinafine and the TISs significantly inhibited most of the tested fungi, with inhibition rates over 80% for *V. mali*, *B. cinerea*, *P. oryzae*, *A. solani*, *F. graminearum*, and *S. sclerotiorum*. The salt formation did not affect the antifungal activity of terbinafine. Figure S17 presents the median effective concentrations (EC_50_) of terbinafine and its salts for the selected fungi.

Table 4 shows the EC_50_ of terbinafine for *B. cinerea*, *A. solani*, and *F. graminearum* as 0.10 mg/L, 0.07 mg/L, and 0.07 mg/L, respectively, indicating an excellent antifungal activity. Terbinafine had a similar or better antifungal activity than the commercial antifungal agent pyraclostrobin against *P. oryzae*, *A. solani*, *F. graminearum,* and *B. cinerea*.

The fungicidal activity of the terbinafine ionic salts (TISs) was compared with terbinafine, the parent compound. The results showed that the TISs had different levels of activity enhancement compared to terbinafine. For example, TIS 5 improved the antifungal activity against *V. mali* and *A. solani* by 14% and 20%, respectively, compared to terbinafine. However, TIS 5 had the highest effective EC_50_ (6.02 μmol/L) against *R. solani* among the TISs and terbinafine. For *B. cinerea*, TIS 1, TIS 3, and TIS 5 had similar antifungal activities to terbinafine. For *P. oryzae*, the EC_50_ of TISs 1–5 (1.1–1.84 μmol/ L) were higher than that of terbinafine, suggesting that adding anions did not increase the activity. For *F. graminearum*, TIS 4 had the best performance among the TISs with a 16% higher bioactivity. TIS 3 had 24% better activity against *R. solani* than terbinafine. These results indicate that the structure of the counter ions added to terbinafine can affect the antifungal activity differently depending on the fungus type.

This study’s findings on the antifungal activity of terbinafine and the TISs against plant pathogens aligned with previous reports on their inhibitory effects against various fungi [5,7,26]. Terbinafine impeded ergosterol biosynthesis by inhibiting squalene epoxidase [26], employing a mechanism different from azole antifungals. This distinction suggests that terbinafine may present a lesser risk of cross-resistance with azoles and other agents [27]. Moreover, terbinafine exhibited fungicidal effects on most fungi, while azoles were primarily fungistatic [26]. These attributes position terbinafine as a potential candidate for the development of innovative agrofungicides. However, comprehensive studies on its effectiveness against major plant diseases and its environmental safety are necessary to ascertain its full value.

### 2.5. In Vivo Activity

**Figure 3:**
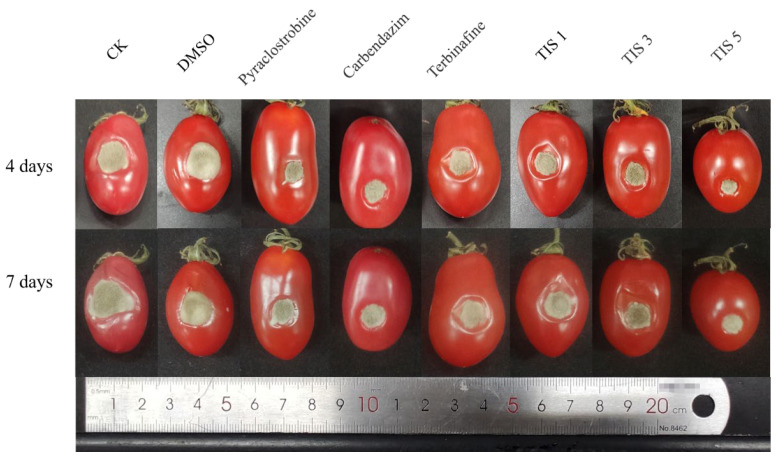
In vivo fungicidal activities against *B. cinerea* on cherry tomatoes.

Based on their prior in vitro antifungal activity against *B. cinerea*, terbinafine, TIS 1, TIS 3, and TIS 5 were chosen for in vivo activity testing on cherry tomatoes at a concentration of 20 mg/L. As shown in Table S2 and Figure 3, all the tested treatments resulted in reduced infection diameters on the cherry tomatoes compared to the control group. Notably, terbinafine, TIS 1, and TIS 3 produced results equivalent to the positive controls of pyraclostrobin and carbendazim. After seven days of incubation, TIS 5 showed the best result (0.74 cm) among all the tested treatments. It outperformed terbinafine (1.07 cm), TIS 1 (1.10 cm), and TIS 3 (1.02 cm). This better performance might have been due to the synergy of the furfuric acid anion.

The observed in vivo antifungal activity of terbinafine and its ionic salts against *B. cinerea* on cherry tomatoes aligned with the results from the in vitro assays. These substances exhibited an equivalent or superior efficacy to the commercially available fungicides pyraclostrobin and carbendazim at a concentration of 20 mg/L. These findings suggest that terbinafine and its ionic salts could serve as potent agrofungicides for managing *B. cinerea*, a ubiquitous plant pathogen [27]. Furthermore, these agents appeared to hold advantages over pyraclostrobin and carbendazim concerning environmental safety and resistance management, given the latter’s negative impacts on aquatic ecosystems [28], human health [29], and fungal susceptibility [28,30,31]. In contrast, terbinafine and its ionic salts are low-toxicity agents with a minimal resistance to agricultural pathogenic fungi, which specifically target squalene epoxidase in fungi [5,7,26]. 

While these results are promising, there are still challenges to be addressed before repurposing terbinafine as an agrofungicide. One significant challenge in repurposing drugs from pharmaceutical contexts for pesticide applications lies in the potential increased resistance to the established therapeutic drugs used in human medicine. This concern is particularly relevant for terbinafine, an antifungal agent commonly used to treat infections in humans and animals. Indeed, multiple reports have surfaced regarding terbinafine resistance in various dermatophyte species, such as *Trichophyton indotineae* and *Trichophyton rubrum*, which are both associated with conditions such as ringworm and nail infections [32,33,34]. These resistance mechanisms commonly involve mutations in the squalene epoxidase (SE) gene that are targeted by terbinafine and other allylamine antifungals [32,33]. The prospect of cross-resistance to other antifungals with differing modes of action also warrants consideration [34]. Hence, the widespread use of terbinafine in agriculture could unintentionally foster the development of resistant strains, thereby potentially compromising its therapeutic effectiveness in both human and veterinary medicine. Consequently, it becomes essential to pursue further research assessing the prevalence and impact of terbinafine resistance in fungi, and to design strategies to preempt or control its emergence and propagation.

## 3. Materials and Methods

### 3.1. Chemicals and Instrumentation 

The chemicals used in this study were procured from various sources. Terbinafine with a purity of 99% was obtained from Shanghai Bide Pharmatech Ltd. (Shanghai, China), while salicylic acid with a purity of 99.5% was sourced from Shanghai Aladdin Biochemical Technology Co., Ltd. (Shanghai, China). Chlorendic acid (98.2% pure) was purchased from Quzhou Rundong Chemical Technology Co., Ltd. (Quzhou, China). and 2-Furoic acid with a 99.5% purity came from Maya Reagent Co., Ltd. (Jiaxing, China). Both maleic acid and fumaric acid, each with a purity of 99.0%, were acquired from Shanghai Macklin Biochemical Co., Ltd. (Shanghai, China). Pyraclostrobin (98%) was provided by Zhejiang Tianyi Biotechnology Co., Ltd. (Shaoxing, China). A range of analytical-grade reagents, including methanol, ethanol, dimethyl sulfoxide (DMSO), acetonitrile, acetone, ethyl acetate, chloroform, toluene, and n-hexane, were procured from Sinopharm Chemical Reagent Co., Ltd. (Beijing, China). The petroleum ether, another analytical-grade reagent, was purchased from Shanghai Titan Scientific Co., Ltd. (Shanghai, China). Ultrapure water was prepared in house using a Milli-Q water purification system (Millipore, Billerica, MA, USA).

The instruments and analytical methods were meticulously chosen to ensure an accurate and precise characterization of the compounds being examined. The ^1^H NMR and ^13^C NMR spectra were recorded using a Bruker Ascend^TM^ 500 MHz NMR spectrometer (Bruker, Germany). The infrared (IR) spectra were obtained using an IRTracer-100 infrared spectrometer (Shimadzu, Japan). A WRS-2C apparatus from Shanghai Precision Instrument Co., Ltd. (Shanghai, China) was used to determine the melting points. The surface tension measurements were conducted utilizing a JYW-200B instrument from Chengde Superior Testing Instrument Co., Ltd. (Chengde, China).

The critical micelle concentrations (CMCs) of all the TISs were determined using an electrical conductivity meter (Leica Desktop Conductivity Meter DDSJ-308F, Shanghai Oustor Industrial Co., Ltd. Shanghai, China). A vacuum-freezing dryer (LC-10N-50C, Shanghai Lichenbangxi Instrument Technology Co., Ltd. Shanghai, China) was employed for the water removal from the prepared compounds. Chromatographic analyses were conducted using an Agilent 1100 HPLC chemical workstation equipped with a UV detector and a Venusil XBP C18 column (250 mm × 4.6 mm, 5 μm). The mobile phase was composed of eluent A (0.1% phosphoric acid solution) and eluent B (methanol), with a gradient elution conducted as follows: 0–10 min, 30% A. The flow rate was set to 0.8 mL/min, and the injection volume was 10 μL. All the samples were filtered through a 0.22 μm nylon membrane filter before analysis. The column was maintained at an ambient temperature, and the detection wavelength was set to 221 nm.

### 3.2. Preparation of Terbinafine Salts

Through acid–base reactions, five terbinafine salts were synthesized. Terbinafine (5 mmol) was dissolved in ethanol, followed by the addition of organic acid ethanol solutions (5 mmol) gradually. The mixed solutions were stirred at room temperature for an hour. Next, the resulting clear solution was evaporated under reduced pressure. While stirring, a petroleum ether (50 mL) was added to the concentrated oily liquid. The mixture was filtered and washed with the petroleum ether and then dried through a 24 h vacuum freeze-drying process.

(E)-N,6,6-trimethyl-N-(naphthalen-1-ylmethyl)hept-2-en-4-yn-1-aminium 2-hydroxybenzoate, **TIS 1**.

White solid (1.68 g, 78%); mp: 102.1–104.7 °C. ^1^H NMR (500 MHz, Chloroform-d) δ 8.16 (d, J = 8.4 Hz, 1H), 7.94–7.87 (m, 3H), 7.70–7.65 (m, 1H), 7.61–7.45 (m, 3H), 7.35 (ddd, J = 8.6, 7.2, 1.8 Hz, 1H), 6.93 (dd, J = 8.2, 1.1 Hz, 1H), 6.84 (td, J = 7.5, 1.2 Hz, 1H), 6.18 (dt, J = 15.8, 7.3 Hz, 1H), 5.81 (dd, J = 15.7, 1.4 Hz, 1H), 4.51 (s, 2H), 3.66 (dd, J = 7.3, 1.4 Hz, 2H), 2.55 (s, 3H), 1.24 (s, 9H). ^13^C NMR (126 MHz, Chloroform-d) δ 173.78, 160.71, 132.88, 132.58, 131.28, 130.05, 129.73, 129.30, 129.20, 128.03, 126.48, 126.02, 125.22, 124.32, 122.24, 117.90, 117.18, 116.06, 115.70, 100.61, 75.13, 56.68, 54.28, 38.25, 29.74, 26.94.

(E)-N,6,6-trimethyl-N-(naphthalen-1-ylmethyl)hept-2-en-4-yn-1-aminium (1S,4R)-3-carboxy-1,4,5,6,7,7-hexachlorobicyclo [2.2.1]hept-5-ene-2-carboxylate, **TIS 2**.

White solid (2.97 g, 87%). ^1^H NMR (500 MHz, DMSO-d6) δ 8.26 (dd, J = 8.1, 1.6 Hz, 1H), 7.93 (dd, J = 7.6, 1.9 Hz, 1H), 7.87 (d, J = 8.0 Hz, 1H), 7.61–7.43 (m, 4H), 6.07 (dt, J = 15.9, 6.7 Hz, 1H), 5.76 (dt, J = 15.9, 1.5 Hz, 1H), 4.02 (s, 2H), 3.24 (d, J = 6.7 Hz, 2H), 2.19 (s, 3H), 1.20 (s, 9H). ^13^C NMR (126 MHz, DMSO-d6) δ 168.30, 138.30, 133.92, 133.65, 132.37, 131.82, 128.82, 128.64, 128.32, 126.42, 126.24, 125.76, 125.01, 114.07, 103.68, 99.28, 80.17, 77.66, 58.82, 58.79, 54.86, 41.60, 31.18, 27.97.

(E)-N,6,6-trimethyl-N-(naphthalen-1-ylmethyl)hept-2-en-4-yn-1-aminium (Z)-3-carboxyacrylate, **TIS 3**.

White solid (1.95 g, 96%); mp: 140.7–142.5 °C. ^1^H NMR (500 MHz, Chloroform-d) δ 8.09 (d, J = 8.5 Hz, 1H), 7.93 (dd, J = 17.3, 8.1 Hz, 2H), 7.69 (d, J = 6.9 Hz, 1H), 7.65–7.59 (m, 1H), 7.58–7.48 (m, 2H), 6.30 (s, 2H), 6.10 (dt, J = 15.2, 7.4 Hz, 1H), 5.91 (d, J = 15.7 Hz, 1H), 4.66 (s, 2H), 3.82 (s, 2H), 2.66 (s, 3H), 1.25 (s, 9H). ^13^C NMR (126 MHz, Chloroform-d) δ 168.48, 134.64, 132.93, 131.05, 130.15, 129.91, 128.30, 127.00, 126.54, 125.57, 124.41, 124.13, 121.67, 120.34, 102.06, 74.74, 56.60, 53.85, 37.81, 29.67, 27.01.

(E)-N,6,6-trimethyl-N-(naphthalen-1-ylmethyl)hept-2-en-4-yn-1-aminium (E)-3-carboxyacrylate, **TIS 4**.

White solid (1.48 g, 73%); mp: 162.2–164.1 °C. ^1^H NMR (500 MHz, DMSO-d6) δ 8.25 (d, J = 7.8 Hz, 1H), 7.97–7.89 (m, 1H), 7.86 (dd, J = 7.3, 1.7 Hz, 1H), 7.53 (pd, J = 6.7, 1.4 Hz, 2H), 7.49–7.42 (m, 2H), 6.64 (s, 2H), 6.07 (dt, J = 15.8, 6.6 Hz, 1H), 5.75 (d, J = 15.9 Hz, 1H), 3.94 (s, 2H), 3.18 (d, J = 6.5 Hz, 2H), 2.16 (s, 3H), 1.20 (s, 9H). ^13^C NMR (126 MHz, DMSO-d6) δ 166.64, 139.11, 134.58, 134.41, 133.94, 132.38, 128.76, 128.41, 127.93, 126.29, 126.17, 125.70, 125.10, 113.44, 98.99, 77.71, 59.32, 59.00, 41.90, 31.19, 27.95.

(E)-N,6,6-trimethyl-N-(naphthalen-1-ylmethyl)hept-2-en-4-yn-1-aminium furan-2-carboxylate, **TIS 5**.

White solid (1.66 g, 82%); mp: 79.5–81.2 °C. ^1^H NMR (500 MHz, Chloroform-d) δ 8.20 (d, J = 8.4 Hz, 1H), 7.86 (t, J = 7.3 Hz, 2H), 7.66 (d, J = 6.9 Hz, 1H), 7.60–7.54 (m,1H), 7.53–7.43 (m, 3H), 7.13 (d, J = 3.4 Hz,1H), 6.47 (dd, J = 3.4, 1.7 Hz, 1H), 6.18 (dt, J = 15.5, 7.2 Hz, 1H), 5.75 (d, J = 15.8 Hz, 1H), 4.49 (s, 2H), 3.59 (d, J = 7.1 Hz, 2H), 2.50 (s, 3H), 1.23 (s, 9H). ^13^C NMR (126 MHz, Chloroform-d) δ 162.82, 147.44, 143.65, 132.86, 132.22, 131.46, 128.64, 128.51, 128.20, 127.84, 125.68, 124.97, 124.31, 122.64, 116.24, 114.50, 110.41, 99.64, 75.43, 56.89, 54.99, 38.74, 29.81, 26.90.

### 3.3. Solubility

This investigation details how to determine the solubility of terbinafine and its salts, guided by the experimental protocol in Vogel’s Textbook of Practical Organic Chemistry [35]. Based on the Snyder polarity index values of the representative solvents, each solvent was tested for its ability to dissolve the target analytes. The solubility data was classified into three categories based on the observed extent of dissolution. Notably, category 1 indicated a favorable degree of solubility, whereby terbinafine and its salts readily dissolved entirely in 1 mL of the solvent. Category 2 denoted a limited solubility, where the target compounds exhibited solubility within 3 mL of the solvent, but not within 1 mL. Category 3 indicated poor solubility, where the target compounds failed to dissolve within 3 mL of the solvent. The experimental procedures were performed at 25 °C and under ambient pressure.

The potential efficacy of pesticide formulations is significantly impacted by the low aqueous solubility of active ingredients [36,37]. To evaluate this, the water solubility of terbinafine and its salts was assessed using high performance liquid chromatography (HPLC) at 25 °C. For this, terbinafine and its salts were added to 200 mL of water and sonicated until a saturated solution were attained. This mixture was maintained in a constant temperature water bath at 25 °C for 15 min, after which it was filtered to produce a saturated aqueous solution of terbinafine and TISs. The filtrate was then passed through a 0.22 μm nylon membrane filter and subjected to HPLC analysis. Based on Wang’s report [38] about the chromatographic parameters, modifications to the mobile phase ratio were made. The replicability of the results was ensured by performing each measurement in triplicate.

### 3.4. Surface Tension

The surface tensions of terbinafine and its salts were evaluated using the Du Noüy ring method [39] at 25 °C. This method involved adjusting the stage positioning of the solvent container such that a platinum ring could be immersed below the liquid interface or pulled through the meniscus. The stage was gradually lowered until the meniscus detached from the ring, indicating that the meniscus volume had reached its maximum. The maximum force was recorded as the surface tension of the solvent. To ensure accuracy, the platinum ring was thoroughly cleaned between each measurement through rinsing with double-distilled water and wiping it dry. The surface tension determination was performed in quadruplicate.

### 3.5. In Vitro Assays of Fungicidal Activities

The fungicidal activities of terbinafine and the TISs were measured using a hyphae growth velocity assay [40] on a potato dextrose agar medium (PDA, Aobox). Eight fungal strains were used: *Valsa malicola* (*V. mali*), *Pythium aphanidermatum* (*P. aphanidermatum*), *Phomopsis capsici* (*P. capsica*), *Botrytis cinerea* (*B. cinerea*), *Rhizoctonia solani* (*R. solani*), *Fusarium graminearum* (*F. graminearum*), *Pyricularia oryzae* (*P. oryzae*), and *Alternaria solani* (*A. solani*). These fungi cause common plant diseases, which were from the Seed Pathology and Fungicide Pharmacology Laboratory at the China Agriculture University.

The fungicidal activity of terbinafine and the TISs at 50 mg/L were screened, which is a standard fungicide volume for pesticide bioactivity tests. If the fungicides had growth inhibition rates over 80% at 50 mg/L, their median lethal concentration (EC_50_) were measured further. The concentrations of 12.50, 6.25, 3.13, 1.56, 0.78, and 0.39 mg/L were used for most of the fungi. For *B. cinerea* and *F. graminearum*, which were more sensitive to terbinafine and the TISs, lower concentrations of 1.56, 0.78, 0.39, 0.20, 0.10, and 0.05 mg/L were applied.

The negative control was a PDA medium with water, and the positive control was a medium with pyraclostrobin and salicylhydroxamic acid [41]. Each treatment was performed three times and measured when the mycelium grew to 5–6 cm long. The growth inhibition rate was calculated as follows.

Inhibitory rate (%) = (Dc − Dt)/Dc × 100%

where Dc is the control colony diameter and Dt is the treated colony diameter.

### 3.6. In Vivo Experiment

The in vivo antifungal bioassays were conducted following Lin’s [42] and Li’s [43] methods. Cherry tomatoes were procured from a local supermarket. These were thoroughly washed with water and disinfected with a 75% ethanol solution for 1 min. Then, the tomatoes were placed on a plate with sterilized gauze and allowed to dry.

Small holes in the tomatoes were made with an inoculating needle and a 5 mm diameter mycelial plug was placed on the skin of each tomato. The inoculated tomatoes were laid on a plate with sterilized gauze and incubated at 25 °C under 100% relative humidity for 24 h. Then, the tomatoes were treated with the selected compounds—terbinafine, TIS 1, TIS 3, and TIS 5—that had good activity in the in vitro antifungal assays. Carbendazim and pyraclofosfom were selected as positive controls and 0.4% DMSO aqueous solution was used as a blank control. Each tomato was soaked in approximately 20 ml of a 20 mg/L solution of the compound for 2 min, with eight replicates for each sample.

Following treatment, the tomatoes were further incubated under the same conditions for an additional 7 days. The diameters of the fungal colonies on the tomato surface were measured using the crossover method on the fourth and seventh days after treatment.

### 3.7. Statistical Analysis

Data analyses were conducted using the statistical analysis software (SPSS, Standard Version 22.0, SPSS Inc., Chicago, IL, USA) to calculate the EC_50_. Three variables—the concentration, inhibition rate, and total—were established before the concentration and the corresponding inhibition rate in the concentration gradient were entered. Then, a probit analysis was performed. A logarithmic base of 10 for the transformation and logit for the model were chosen. The EC_50_ value was the concentration value corresponding to an odds ratio of 0.5.

## 4. Conclusions

To summarize, five ionic salts of terbinafine were synthesized and characterized and evaluated for their potential for agricultural use as fungicides. This study illustrated that converting terbinafine into ionic salts can effectively address its low water solubility issue and augment the fungicidal efficacy of the original compound. It was observed that the cationic components significantly influenced the fungicidal activity, whereas the anionic components had a relatively smaller contribution. Certain anions demonstrated synergistic effects, while others showed counter effects, depending on the specific fungus. Overall, the study provides important insights into the potential of terbinafine and its ionic salts as fungicides in agriculture and highlights the need for further research in this field.

The strategy of drug reuse and development from pharmaceutical to pesticide applications can significantly shorten the time and cost of drug development. Transforming drugs into salts is an effective strategy to enhance their physical and chemical properties without altering the drug itself. The versatility of the drug can be increased using a designable counterion, improving its practical application. However, certain prerequisites and limitations apply to the formation of drug salts. First and foremost, the drug in question must be either acidic or basic, and it should maintain stability when exposed to counter ions via an acid or base. This stipulates that the ΔpKa of the relevant acid or base must exceed three. Not all drugs fulfill this criterion, which consequently restricts the assortment of drugs that can undergo salt transformation for this purpose.

In terms of future perspectives, it is believed that it would be worthwhile to further explore how different counterions can influence the properties of the salt-form drugs, especially regarding their stability, solubility, and overall antifungal activity. Additionally, investigations into other strategies to improve the physical and chemical properties of pharmaceuticals, beyond salt formation, could open up new avenues for the development of efficient and cost-effective pesticides. Lastly, further in-depth studies on the potential resistance to these repurposed drugs and how it can be managed effectively would also be beneficial to ensure their long term efficacy in agricultural use.

## Figures and Tables

**Table 1:** The chemical shift of the hydrogen on the carbon attached to the tertiary amine.

API Combination	N-CH_3_ (ppm)	N-CH_2_-Ar (ppm)	N-CH_2_-CH (ppm)
TIS 1 (CDCl_3_)	2.51	4.51	3.66
TIS 2 (DMSO)	2.19	3.98	3.24
TIS 3 (CDCl_3_)	2.66	4.66	3.82
TIS 4 (DMSO)	2.16	3.94	3.18
TIS 5 (CDCl_3_)	2.5	4.49	3.59
Terbinafine (CDCl_3_)	2.21	3.88	3.12
Terbinafine (DMSO)	2.11	3.86	3.1

**Table 2:** Solubilities of the prepared TISs 1–5 at 25 °C.

Salt	Water	Methanol	DMSO	Acetonitrile	Acetone	Isopropanol	Ethyl acetate	Chloroform	Toluene	*n*-Hexane
	9.0 ^a^	6.6	6.5	6.2	5.1	4.3	4.3	4.1	2.3	0.0
T	− ^b^	+ ^c^	+	+	+	+	+	+	+	+
TIS 1	−	+	+	+	+	−	+	+	+	−
TIS 2	−	−	+	−	−	−	−	−	−	−
TIS 3	−	+	+	−	−	−	−	+	−	−
TIS 4	−	−	+	−	−	−	−	−	−	−
TIS 5	−	+	+	+	+	+	+	+	+	−

^a^ Snyder polarity index; ^b^ “−” means insoluble in a 3 mL solvent, poor solubility, displayed with a red background; ^c^ “+” means soluble in a 1 mL solvent, good solubility, displayed here with a green background; “±” means soluble in a 3 mL solvent, limited solubility. The test amount was 0.1 g.

**Table 3:** In vitro fungicidal activities (inhibition rate) of terbinafine and its salts at 50 mg/L.

Drug	Inhibition Rate at 50 mg/L (%)							
	*V. m*	*B. c*	*P. o*	*P. c*	*A. s*	*R. s*	*F. g*	*P. a*
T	100 ± 0	100 ± 0	99.64 ± 0.36	48.75 ± 0.45	100 ± 0	84.21 ± 0.53	100 ± 0	68.21 ± 1.87
TIS 1	100 ± 0	100 ± 0	99.05 ± 0.21	46.97 ± 1.18	100 ± 0	84.79 ± 1.25	100 ± 0	74.61 ± 0.38
TIS 2	100 ± 0	100 ± 0	96.09 ± 0.36	43.85 ± 1.61	98.8 ± 0	80.75 ± 0.72	100 ± 0	69.65 ± 1.7
TIS 3	100 ± 0	100 ± 0	99.29 ± 0	33.16 ± 2.52	100 ± 0	83.4 ± 0.35	100 ± 0	69.54 ± 0
TIS 4	100 ± 0	100 ± 0	98.7 ± 0.54	47.64 ± 1.58	100 ± 0	85.82 ± 1.51	100 ± 0	65.23 ± 0.47
TIS 5	100 ± 0	100 ± 0	96.68 ± 0.42	55.88 ± 1.89	98.67 ± 0.61	82.02 ± 0.91	100 ± 0	71.96 ± 2.68

Values are the mean ± the standard deviation (SD) of three replicates. *V. m: V. mali. B. c: B. cinerea. P. o: P. oryzae. P. c: P. capsica. A. s: A. solani. R. s: R. solani. F. g: F. graminearum. P. a: P. aphanidermatum*.

**Table 4:** In vitro antifungal EC_50_ value against *V. mali*, *B. cinerea*, *P. oryzae*, *A. solani*, *R. solani,* and *F. graminearum* of terbinafine and its salts.

Fungi	Drugs	Regression Equation	r^2^	EC_50_		95% Confidence Intervals (mg/L)
				(mg/L)	(μmol/L)	
*V. m*	Terbinafine	y = 2.62x − 0.22	0.969	1.25	4.28	0.98–1.57
	TIS 1	y = 2.12x − 0.77	0.985	2.24	5.21	1.93–2.57
	TIS 2	y = 2.87x − 1.87	0.955	4.28	6.29	3.16–6.00
	TIS 3	y = 2.15x − 0.66	0.986	2.03	4.98	1.77–2.34
	TIS 4	y = 2.17x − 0.59	0.997	1.88	4.60	1.64–2.14
	TIS 5	y = 2.28x − 0.39	0.986	1.49	3.69	1.30–1.73
	Pyraclostrobin	y = 0.59x + 0.06	0.995	0.79	2.02	0.03–1.71
*B. c*	Terbinafine	y = 3.18x + 3.16	0.976	0.10	0.34	0.09–0.11
	TIS 1	y = 3.03x + 2.6	0.959	0.14	0.32	0.11–0.17
	TIS 2	y = 3.53x + 2.12	0.990	0.25	0.37	0.23–0.28
	TIS 3	y = 3.53x + 3.09	0.942	0.13	0.33	0.10–0.18
	TIS 4	y = 3.7x + 3.07	0.974	0.15	0.36	0.12–0.18
	TIS 5	y = 4.27x + 3.77	0.966	0.13	0.33	0.11–0.15
	Pyraclostrobin	y = 1.88x + 1.74	0.993	0.12	0.31	0.01–0.15
*P. o*	Terbinafine	y = 0.98x + 0.55	0.998	0.28	0.95	0.13–0.43
	TIS 1	y = 1.02x + 0.33	0.991	0.47	1.10	0.28–0.66
	TIS 2	y = 1.15x + 0.07	0.991	0.88	1.29	0.65–1.11
	TIS 3	y = 1.17x + 0.28	0.969	0.58	1.41	0.39–0.76
	TIS 4	y = 1.32x + 0.17	0.993	0.75	1.84	0.57–0.94
	TIS 5	y = 1.1x + 0.34	0.997	0.49	1.22	0.31–0.68
	Pyraclostrobin	y = 0.56x + 0.13	0.991	0.60	1.54	0.25–0.99
*A. s*	Terbinafine	y = 1.25x + 1.43	0.998	0.07	0.25	0.05–0.09
	TIS 1	y = 1.42x + 1.25	0.936	0.13	0.30	0.07–0.19
	TIS 2	y = 0.92x + 0.68	0.987	0.18	0.27	0.13–0.24
	TIS 3	y = 0.99x + 0.83	0.965	0.15	0.36	0.11–0.19
	TIS 4	y = 1.23x + 1.16	0.986	0.11	0.28	0.09–0.14
	TIS 5	y = 1.1x + 1.19	0.996	0.08	0.20	0.06–0.11
	Pyraclostrobin	y = 0.3x + 0.2	0.981	0.23	0.59	0.07–0.56
*R. s*	Terbinafine	y = 1.29x − 0.33	0.961	1.73	5.94	1.41–2.10
	TIS 1	y = 1.8x − 0.78	0.931	2.49	5.81	1.69–3.72
	TIS 2	y = 1.48x − 0.78	0.975	3.32	4.88	2.78–4.03
	TIS 3	y = 1.44x − 0.39	0.984	1.84	4.51	1.53–2.19
	TIS 4	y = 1.49x − 0.56	0.972	2.30	5.65	1.93–2.75
	TIS 5	y = 1.49x − 0.59	0.980	2.43	6.02	2.04–2.90
	Pyraclostrobin	y = 0.55x + 0.62	0.999	0.07	0.19	0.01–0.21
*F. g*	Terbinafine	y = 1.4x + 1.66	0.943	0.07	0.25	0.06–0.09
	TIS 1	y = 1.25x + 1.27	0.988	0.10	0.23	0.07–0.12
	TIS 2	y = 1.37x + 1.08	0.995	0.16	0.24	0.12–0.21
	TIS 3	y = 1.32x + 1.35	0.984	0.10	0.24	0.08–0.12
	TIS 4	y = 1.3x + 1.38	0.995	0.09	0.21	0.06–0.11
	TIS 5	y = 1.33x + 1.29	0.983	0.11	0.27	0.08–0.13
	Pyraclostrobin	y = 0.74x − 0.12	0.993	1.43	3.68	0.91–3.27

*V. m: V. mali; B. c: B. cinerea; P. o: P. oryzae; A. s: A. solani; R. s: R. solani; F. g: F. graminearum*.

## Data Availability

All the data are in the manuscript and Supplementary Materials.

## Supplementary Materials

The following supporting information can be downloaded at: https://www.mdpi.com/article/10.3390/molecules28124722/s1, Figures S1–S15: the ^1^H NMR, ^13^C NMR, and FT-IR spectra of the TISs, Figure S16: the CMC (mM) determination for the prepared TISs at 25 °C, Figure S17: the photos of the in vitro antifungal activities of terbinafine and its salts against (a) *V. mali.*, (b) *B. cinerea.*, (c) *P. oryzae.*, (d) *A. solani.*, (e) *R. solani.*, and (f) *F. graminearum*, Table S1: the Solubility of the prepared TISs in all kinds of solvents at 25 °C, Table S2: the diameter of infection in vivo fungicidal activities against *B. cinerea* on cherry tomatoes.

## Sample Availability

Samples are available from the authors.
