# Supplementary material for: The Effect of Terbinafine and Its Ionic Salts on Certain Fungal Plant Pathogens
Source: Molecules. 2023 Jun 12;28(12):4722. doi: 10.3390/molecules28124722 (PMC10303384; doi:10.3390/molecules28124722)
Supplement: Supplementary file 1 [file molecules-28-04722-s001.zip › molecules-2353270-supplementary.pdf]

Supplementary Materials

The Effect of Terbinafine and Its Ionic Salts on Certain Fungal Plant Pathogens

Tao Wang [1], Qiuxiao Wang [1], Yifei Zhou [1], Yaolin Shi [2] and Haixiang Gao [3,*]

[1] Department of Applied Chemistry, College of Science, China Agricultural University, Beijing 100193, China; wangtao978@cau.edu.cn (T.W.); bs20203100716@cau.edu.cn (Q.W.); zhouyifei@cau.edu.cn (Y.Z.)
[2] College of Materials Science and Engineering, Beijing University of Chemical Technology, Beijing 100029, China; 2020020050@buct.edu.cn
[3] Innovation Center of Pesticide Research, Department of Applied Chemistry, China Agricultural University, Beijing 100193, China
* Correspondence: hxgao@cau.edu.cn

Figure S1. ^{1}H NMR spectrum of **TIS 1.**

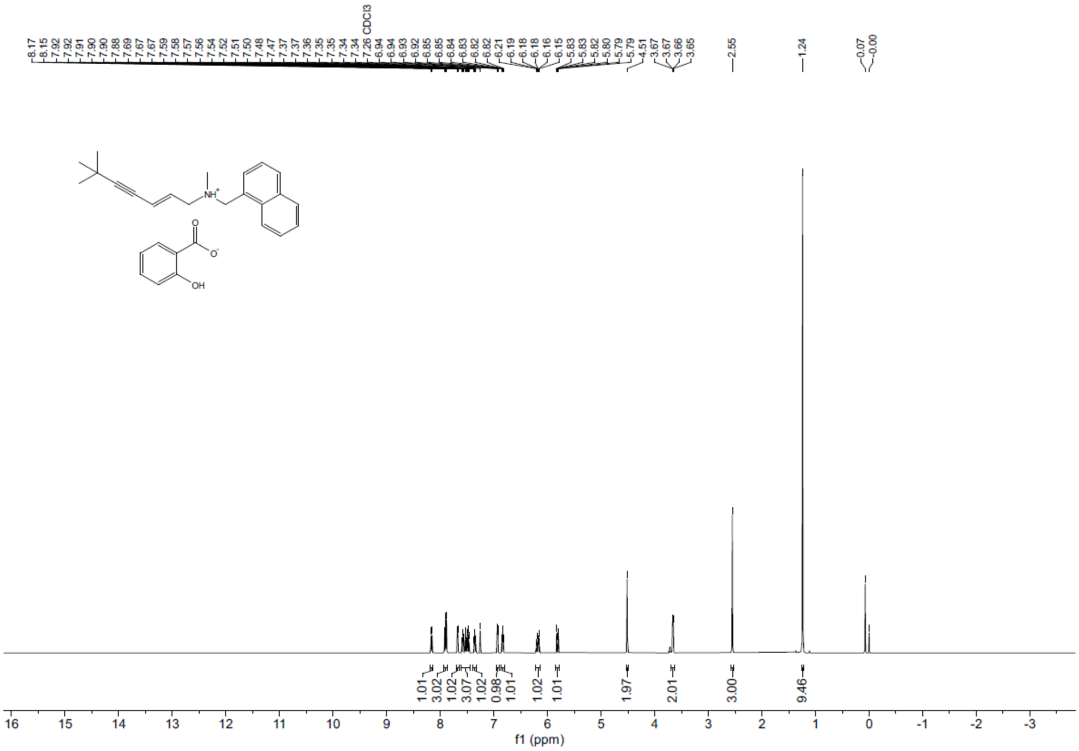

Figure S2. ^{13}C NMR spectrum of **TIS 1.**

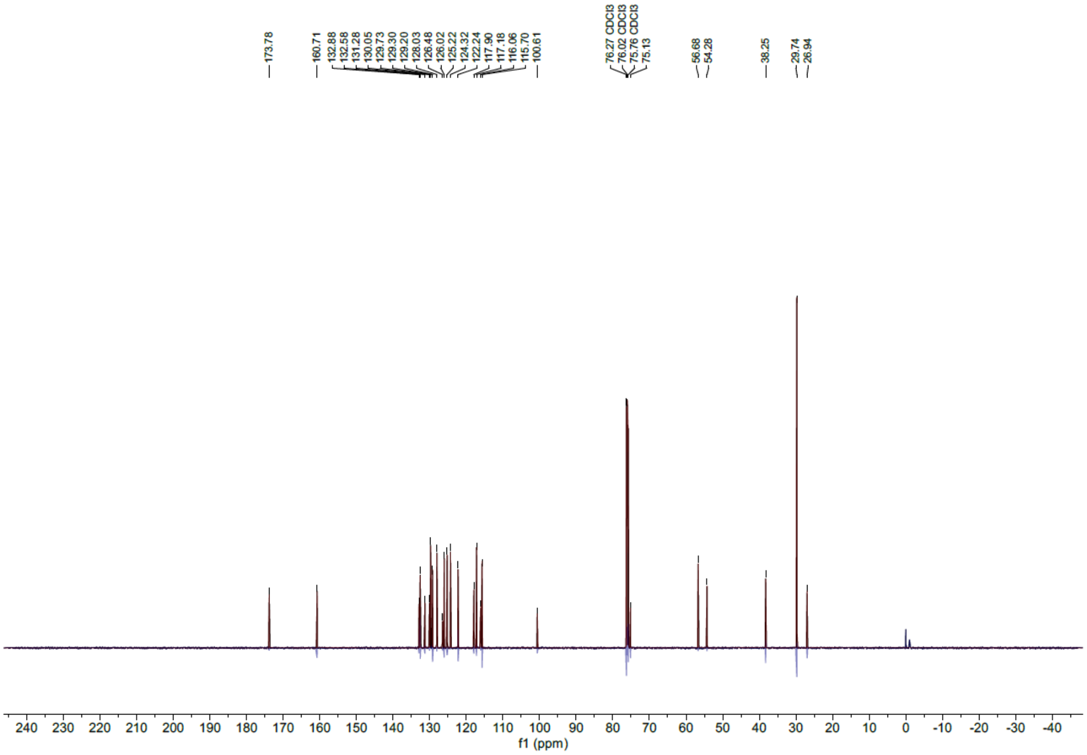

Figure S3. FT-IR spectrum of **TIS 1.**

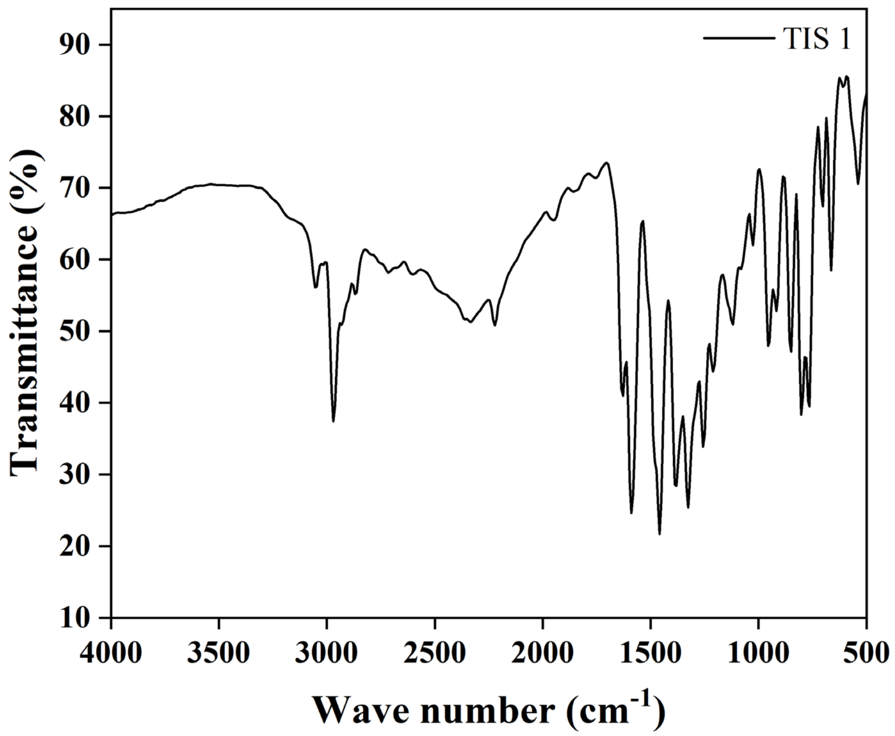

Figure S4. ^{1}H NMR spectrum of **TIS 2.**

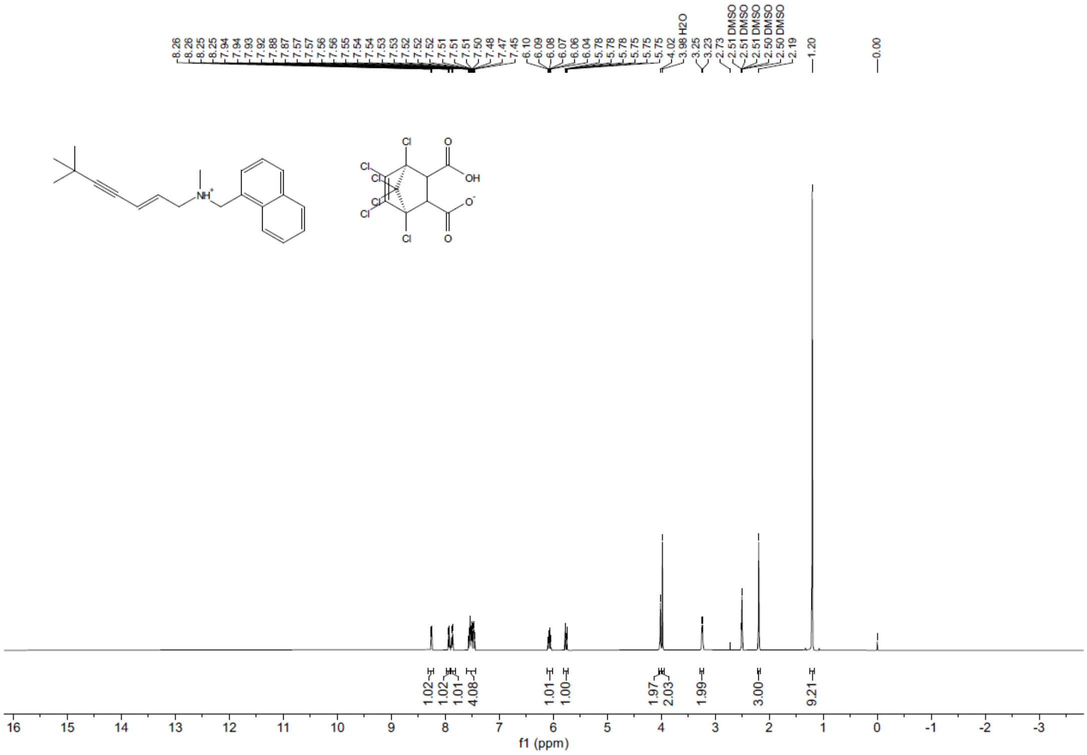

Figure S5. ^{13}C NMR spectrum of **TIS 2.**

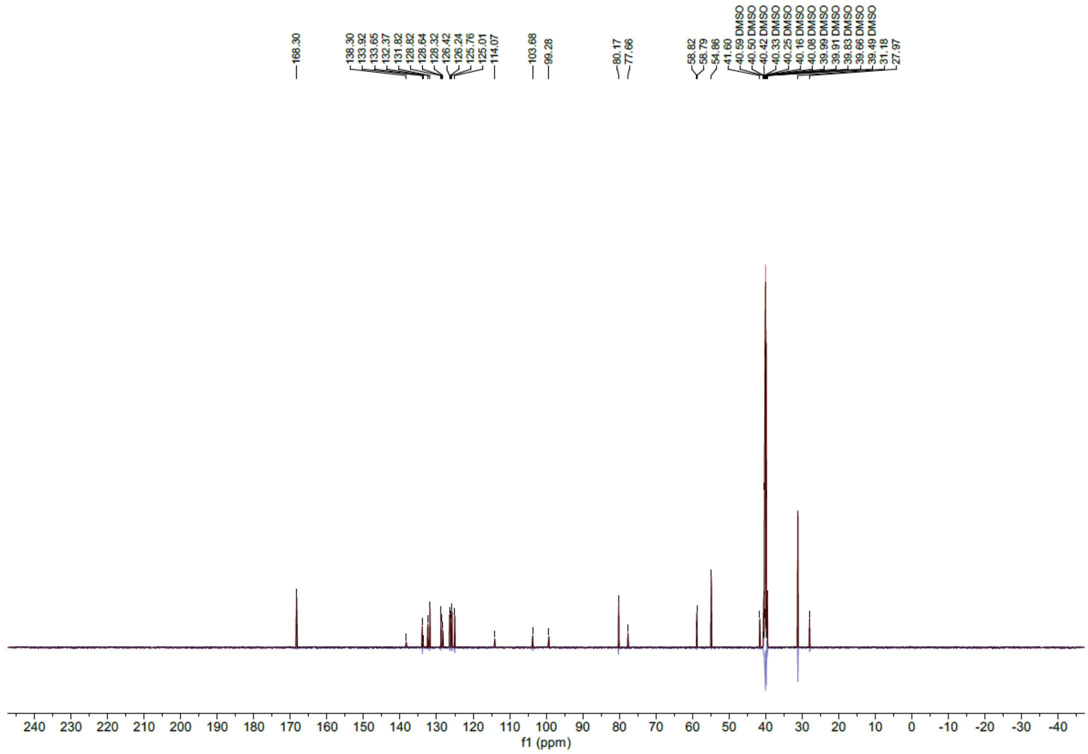

Figure S6. FT-IR spectrum of **TIS 2.**

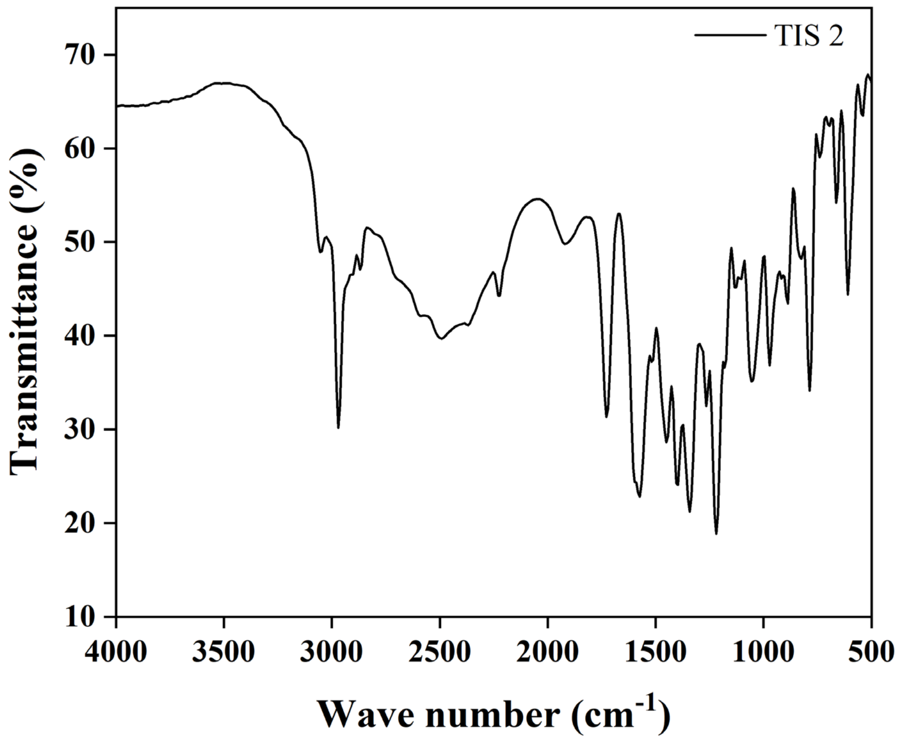

Figure S7. ^{1}H NMR spectrum of **TIS 3.**

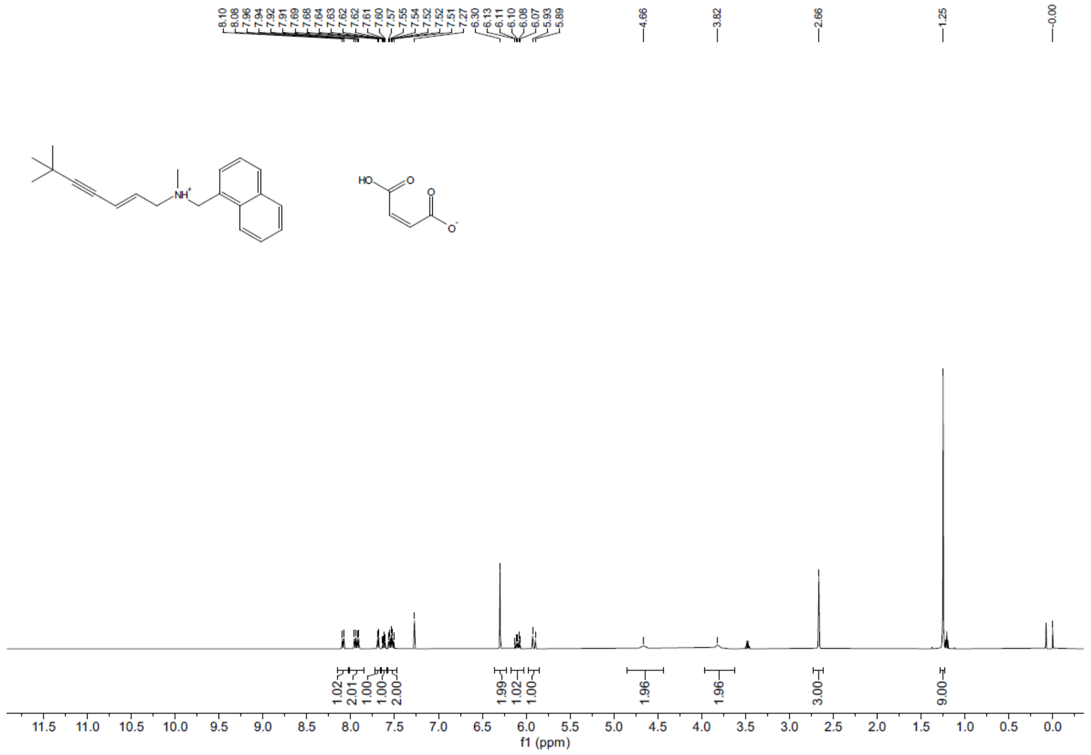

Figure S8. ^{13}C NMR spectrum of **TIS 3.**

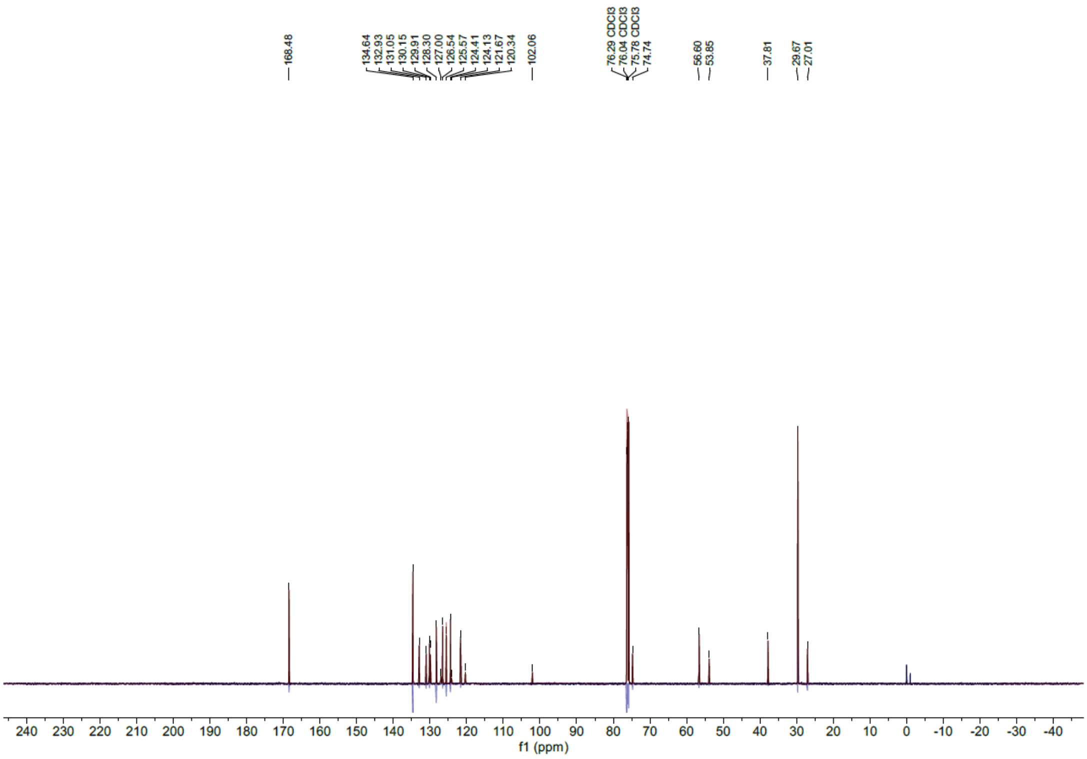

Figure S9. FT-IR spectrum of **TIS 3.**

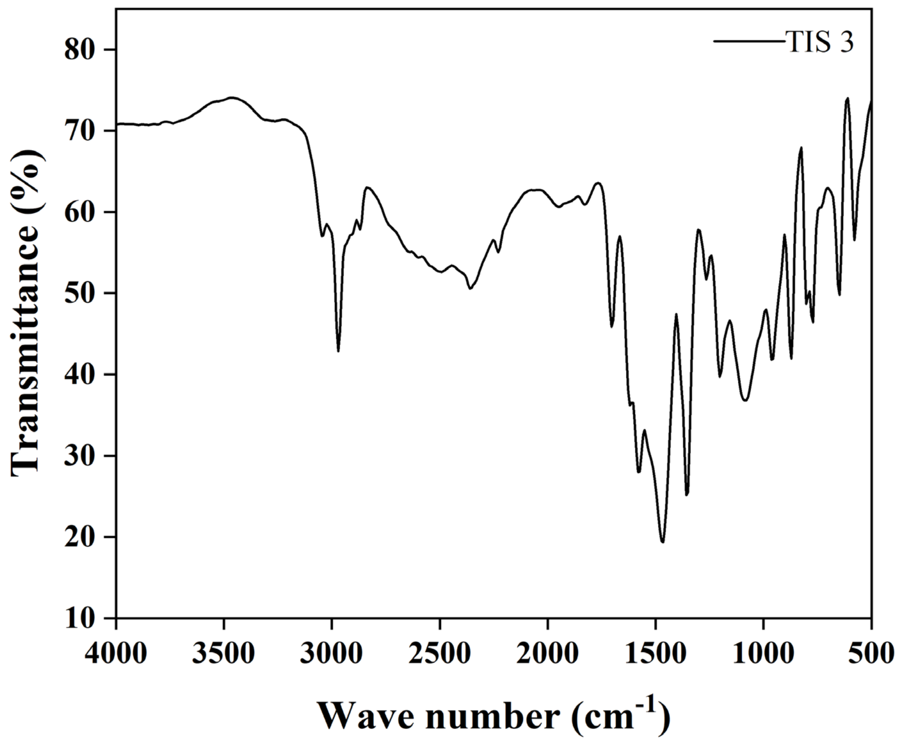

Figure S10. ^{1}H NMR spectrum of **TIS 4.**

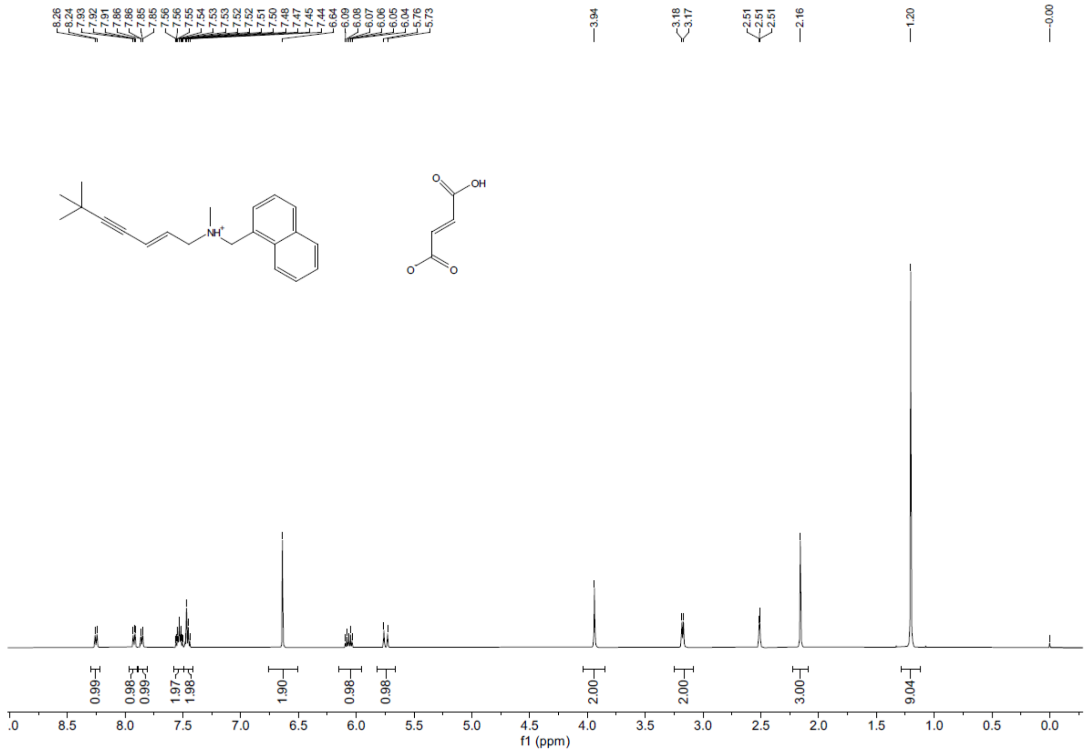

Figure S11. ^{13}C NMR spectrum of **TIS 4.**

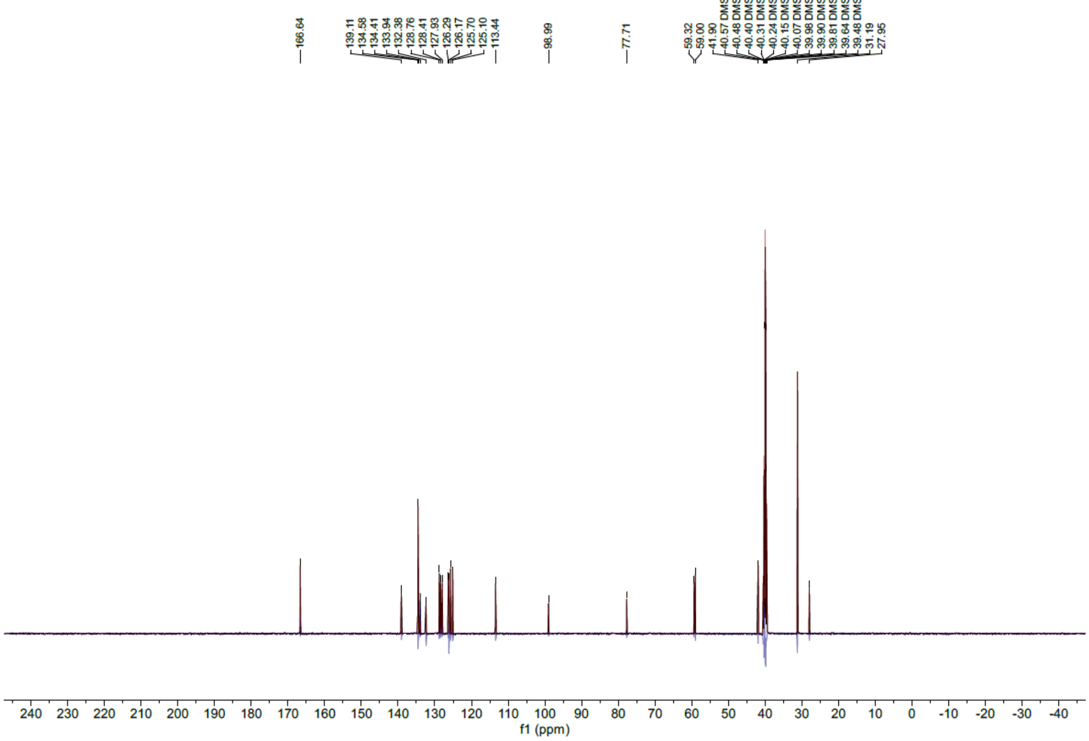

Figure S12. FT-IR spectrum of **TIS 4.**

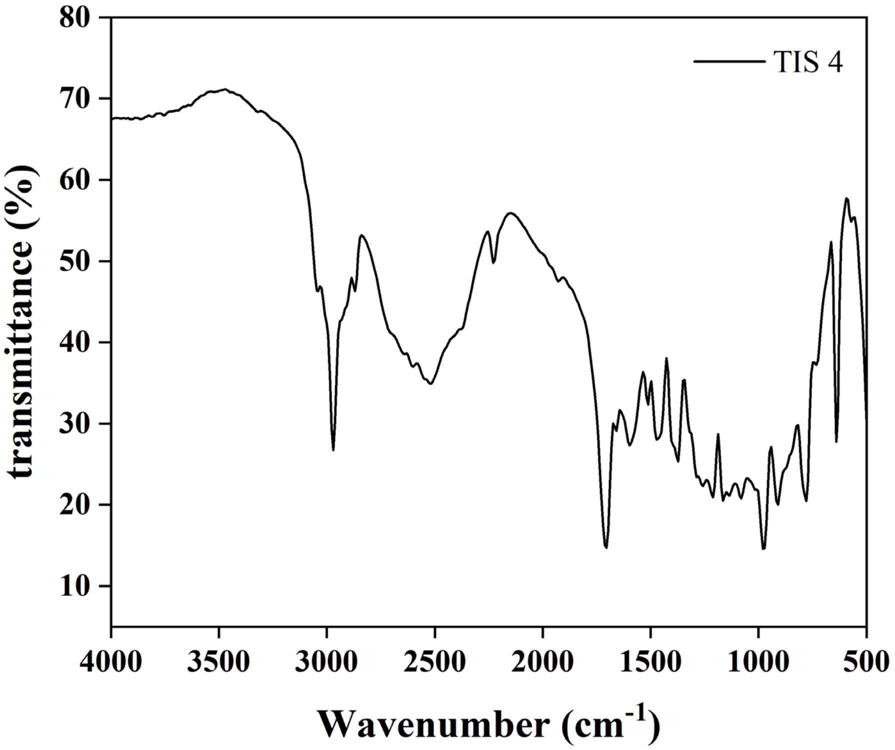

Figure S13. ^{1}H NMR spectrum of **TIS 5.**

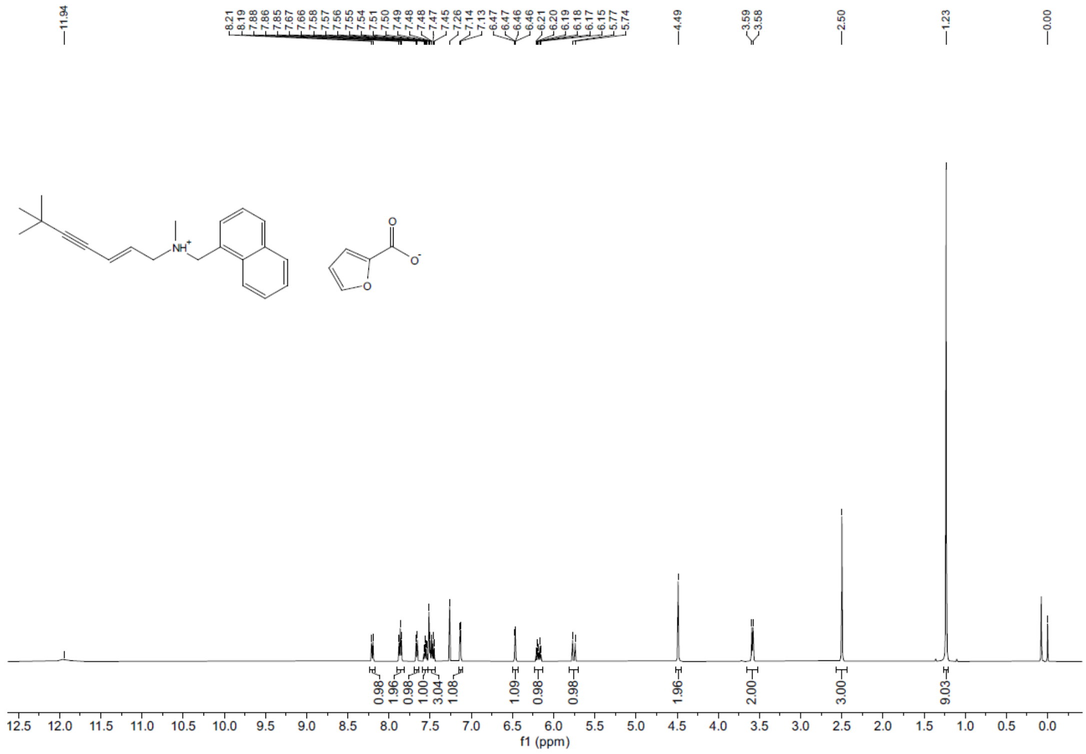

Figure S14. ^{13}C NMR spectrum of **TIS 5.**

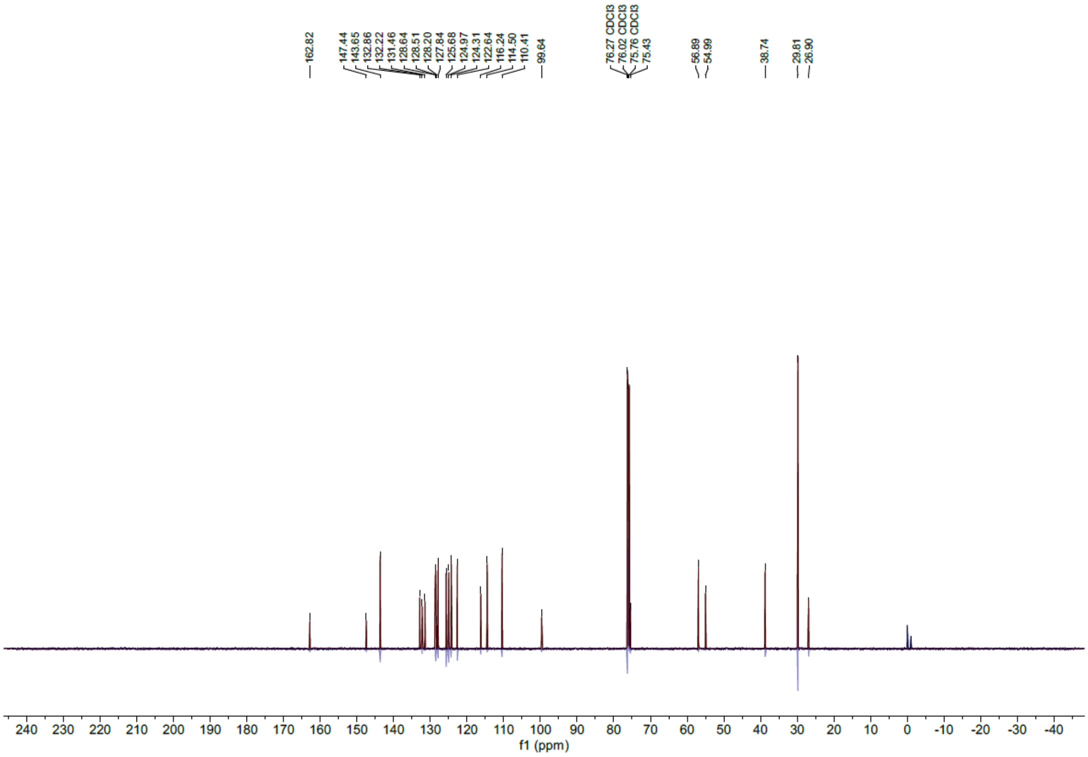

Figure S15. FT-IR spectrum of **TIS 5.**

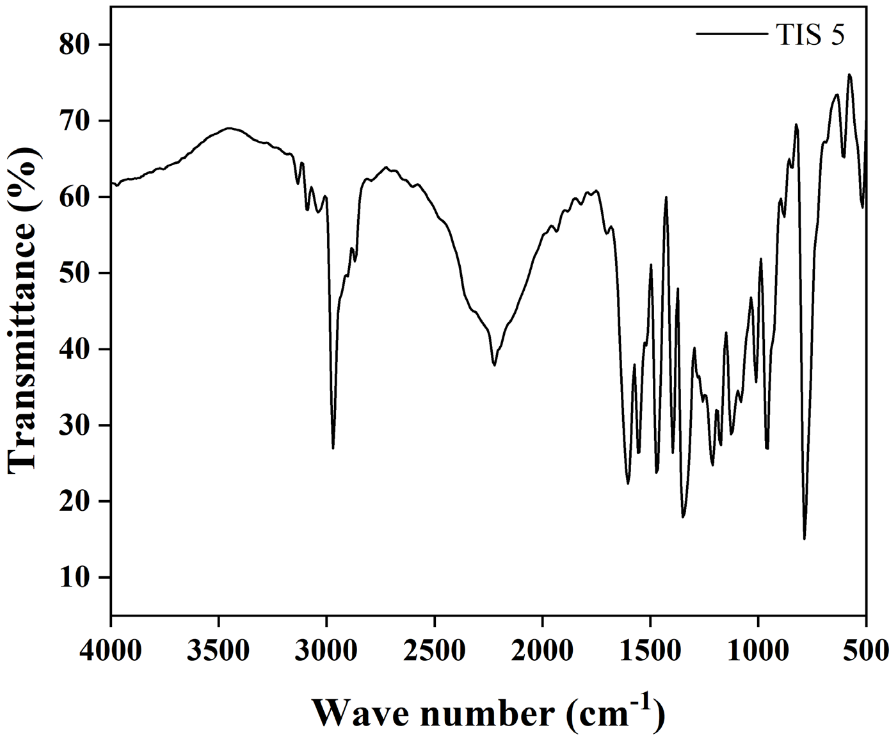

Figure S16. CMC (mM) determination for the prepared TISs, at 25ºC, using distilled water.

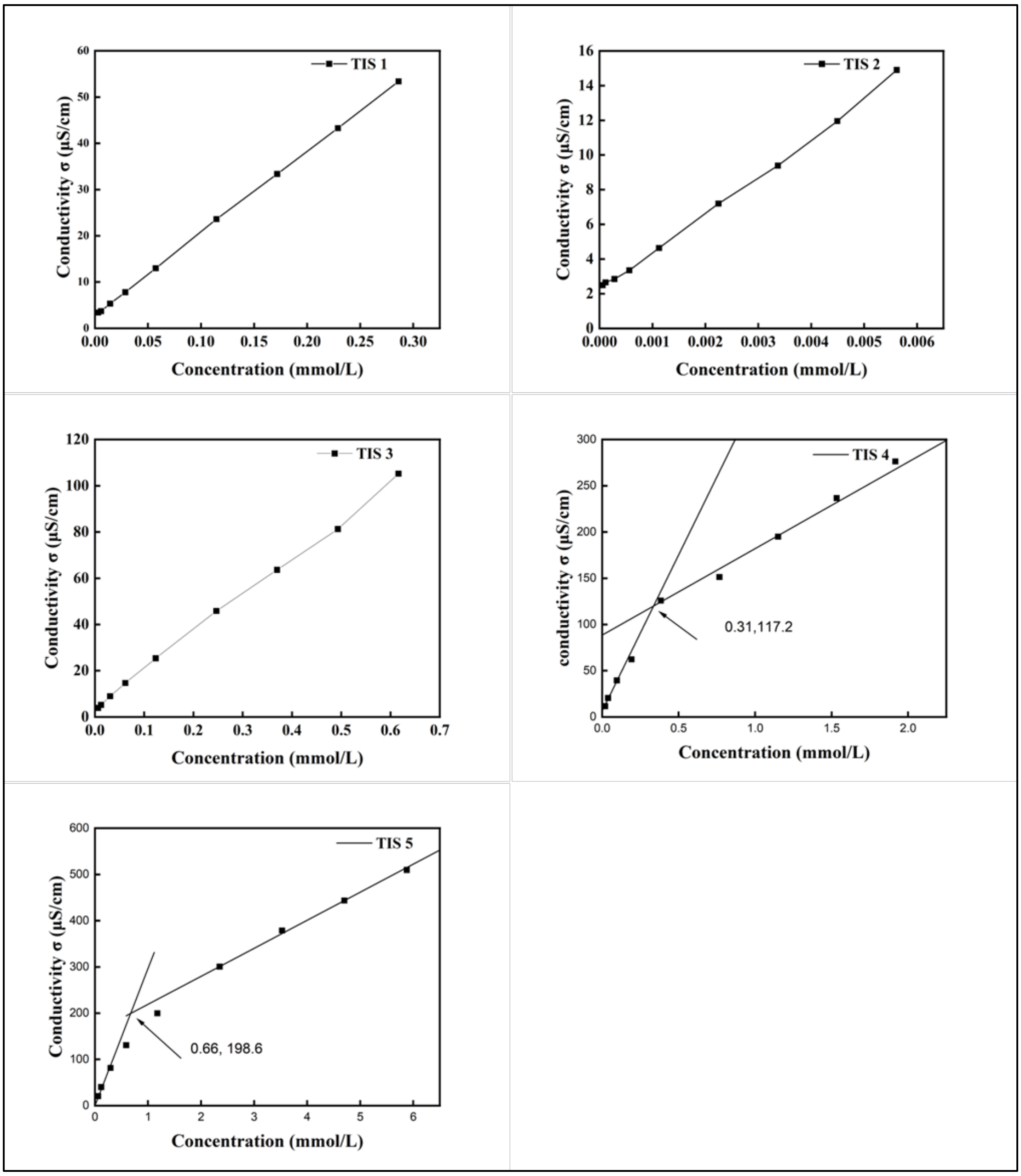

Figure S17. Photos of the *in vitro* antifungal activities of terbinafine and its salts against (a) *V. mali*., (b) *B. cinerea*., (c) *P. oryzae*., (d) *A. solani*., (e) *R. solani*., and (f) *F. graminearum*. Terbinafine and TISs were all dissolved in DMSO.

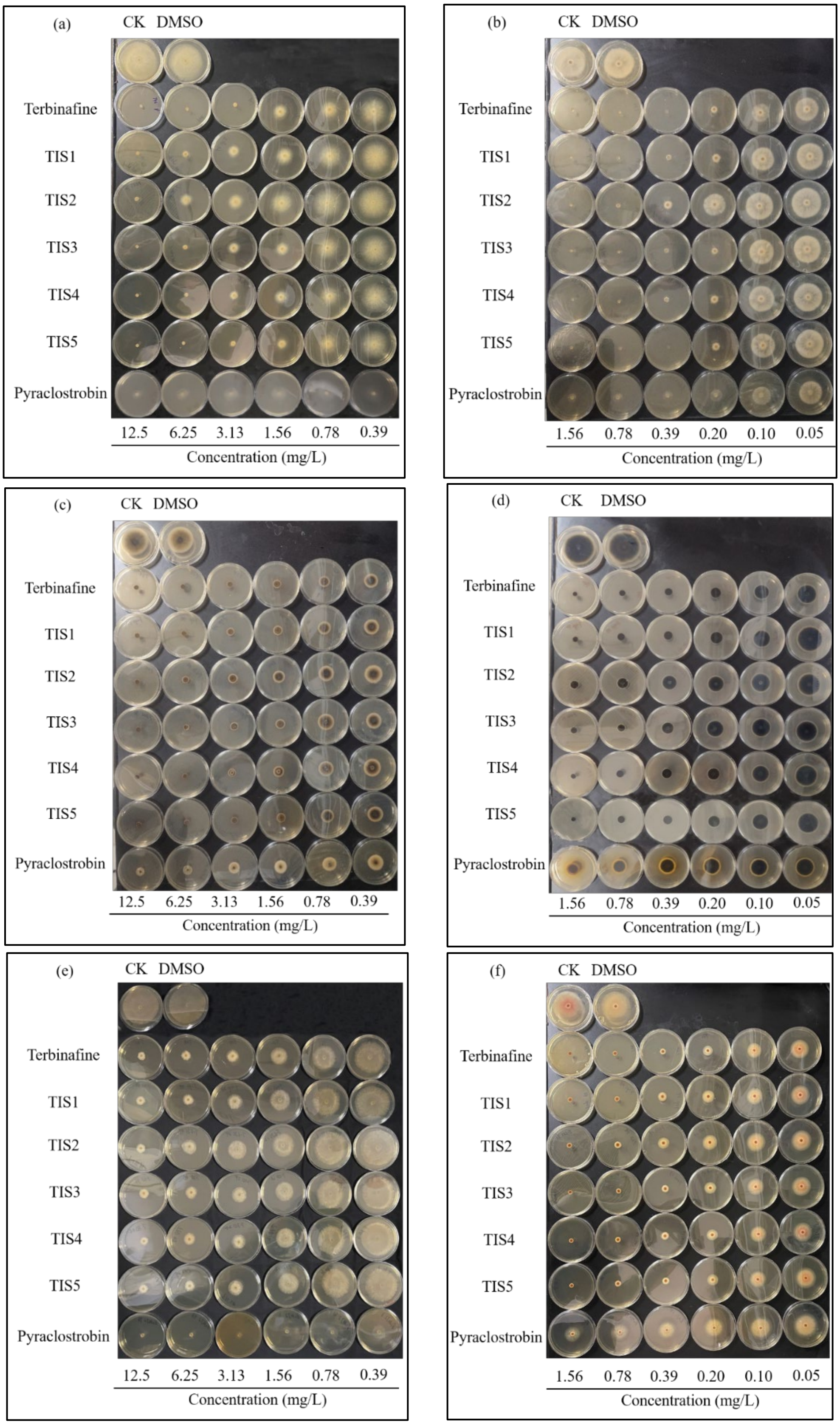

Table S1. Solubility of the prepared TISs in all kinds of solvents at 25 ºC（mol/L）.

Drug mol/L	Water	Methanol	DMSO	Acetonitrile	Acetone	Isopropanol	Ethyl acetate	Chloroform	Toluene	*n*-Hexane
T	5.22E-06									
TIS 1	2.84E-04					5.18E-06				5.02E-07
TIS 2	5.62E-06	5.54E-08		1.55E-07	8.23E-06	7.03E-08	4.56E-07	1.39E-06	3.66E-07	2.21E-08
TIS 3	6.16E-04			1.49E-03	3.76E-06	3.06E-06	4.85E-04		4.21E-05	2.34E-07
TIS 4	1.92E-03	7.75E-03		3.01E-04	2.07E-03	1.92E-03	8.80E-04	3.21E-04	2.61E-07	7.09E-05
TIS 5	5.88E-03									1.02E-06

Table S2. Diameter of infection in vivo fungicidal activities against B. cinerea on cherry tomatoes.

	Diameter of infection (cm)	
	4 days	7 days
Terbinafine	0.89	1.07
TIS 1	0.78	1.10
TIS 3	0.82	1.02
TIS 5	0.61	0.74
CK	1.12	1.63
DMSO	1.24	1.33
Pyraclostrobine	0.71	0.92
Carbendazim	0.85	0.92
